# Morphology and phylogenetic analyses reveal two new species and a new record of *Phaeocollybia* (*Agaricales*, *Hymenogastraceae*) from southwestern China

**DOI:** 10.3897/mycokeys.142.206931

**Published:** 2026-10-27

**Authors:** Rui-Yu Li, Duan-Fen Zhao, Ye-Ting Li, Yuan Luo, E-Xian Li, Han-Bing Song, Hong-Wei Shen, Zong-Long Luo, Song-Ming Tang

**Affiliations:** 1 College of Agriculture and Biological Science, Dali University, Dali 671003, Yunnan, China College of Agriculture and Biological Science, Dali University Dali China; 2 Biotechnology and Germplasm Resources Institute, Yunnan Academy of Agricultural Sciences, Kunming 650205, China Biotechnology and Germplasm Resources Institute, Yunnan Academy of Agricultural Sciences Kunming China; 3 Co-Innovation Center for Cangshan Mountain and Erhai Lake Integrated Protection and Green Development of Yunnan Province, Dali University, Dali 671003, Yunnan, China Co-Innovation Center for Cangshan Mountain and Erhai Lake Integrated Protection and Green Development of Yunnan Province, Dali University Dali China

**Keywords:** Morphology, novel taxa, phylogeny, taxonomy

## Abstract

*Phaeocollybia* is a globally distributed agaricoid genus and remains poorly studied in China. In this study, field surveys were conducted, and specimens of *Phaeocollybia* were collected in Yunnan Province and the Xizang Autonomous Region of southwestern China from 2024 to 2025. Based on detailed morphological observations and multilocus phylogenetic analyses using internal transcribed spacer (ITS), nuclear ribosomal large subunit (nrLSU), and second-largest subunit of RNA polymerase II (*rpb2*) sequence data, two new species and one species newly recorded from China are recognized. *Phaeocollybia dingjieensis* is characterized by a paraboloid to broadly conical pileus with dark moderate orange vertical stripes, limoniform to amygdaliform basidiospores, and the presence of clamp connections. *Phaeocollybia lulangensis* is distinguished by a broadly conical to conical pileus with an oily to subwaxy luster, smooth to rarely verruculose basidiospores, and narrowly cylindrical to lageniform cheilocystidia with long rostrate apices. In addition, *Phaeocollybia attenuata*, previously known only from North America, is reported from China for the first time; its morphological variations and phylogenetic placement are documented. Phylogenetic analyses strongly support the delimitation of these taxa. Comprehensive morphological comparisons with related taxa are provided, and an updated dichotomous key to the 15 species of *Phaeocollybia* currently known from China is provided. These findings significantly expand the known diversity and distribution of *Phaeocollybia* in China.

## Introduction

*Phaeocollybia* R. Heim is an agaricoid genus belonging to *Hymenogastraceae* within *Agaricales*. The genus is morphologically defined by a suite of characters: a typically campanulate to conical, often viscid pileus; a tough, cartilaginous stipe that extends below ground into a long, rooting pseudorhiza; ornamented, apically beaked (amygdaliform to limoniform) basidiospores; a sarcodimitic hyphal system in the tramal tissues; a monovelangiocarpic mode of development; and the presence of diagnostically important, aseptate tibiiform diverticula on the mycelium and remnants of the pellicular veil (Smith 1957; Smith and Trappe 1972; Redhead and Malloch 1986; Norvell 1998a, 1998b; Norvell and Exeter 2007; Norvell et al. 2022). This genus is widely distributed in forests across Europe, Asia, Australia, South America, Central America, and North America and currently comprises 88 accepted species (https://www.catalogueoflife.org/data/search?) (Horak 1980; Redhead and Smith 1986; Horak and Halling 1991; Norvell 2000; Wei et al. 2010; Coimbra et al. 2012; Khan et al. 2016; Norvell et al. 2022). To date, 12 species of *Phaeocollybia* have been documented in China (Smith 1957; Liu 1995; Wei et al. 2010).

Historically, species currently assigned to *Phaeocollybia* were initially placed by Fries (1838) within a heterogeneous assemblage of brown-spored agaricoid fungi in the tribe *Naucoria* of *Agaricus*. This tribe was subsequently elevated to generic rank as *Naucoria* (Fr.) P. Kumm., a concept independently adopted by Kummer (1871) and Quélet (1872). To accommodate morphologically distinct taxa from *Naucoria*, Heim (1931) established *Phaeocollybia*, circumscribing it as a genus characterized by yellow-brown ornamented basidiospores, viscid to glutinous pilei, cartilaginous radicating stipes (pseudorhizae), and gelatinous tissues. Konrad (1934) designated *P. lugubris* (Fr.) R. Heim as the prospective lectotype, a typification later conserved by the IAPT Committee for Fungi (Kuyper and Bas 1986; Gams 1994). At the family level, *Phaeocollybia* was long placed within *Cortinariaceae* based on its brown, ornamented basidiospores lacking a germ pore (Singer 1986). However, multilocus phylogenetic analyses by Matheny et al. (2006), using *P. festiva* (Fr.) R. Heim as a representative taxon, resolved the genus within *Hymenogastraceae*, a placement subsequently supported by additional molecular studies (Norvell et al. 2010, 2022; Kalichman et al. 2020).

Early taxonomic studies of *Phaeocollybia* relied primarily on morphological characters (Smith 1957; Smith and Trappe 1972; Redhead and Malloch 1986; Bandala et al. 1996; Norvell 1998a, 1998b; Norvell and Exeter 2007). Smith (1957) proposed the first infrageneric classification, dividing the genus into two sections, sect. *Phaeocollybia* and sect. *Versicolores*, based on cheilocystidial morphology. Singer (1970) later expanded this framework by adding three additional sections: sect. *Microsporae*, sect. *Radicatae*, and sect. *Subattenuatae*, based on basidiospore size and the presence or absence of clamp connections. Bandala and Montoya (1994) subsequently synthesized these sectional concepts into two subgenera—subgen. *Phaeocollybia* (encompassing sections characterized by the absence of clamp connections) and subgen. *Fibulophaeocollybia* (encompassing sections characterized by their presence). However, the utility of morphological characters is constrained by their inherent plasticity; basidiome size, pileus shape, and color can vary significantly with ontogeny (Hyde et al. 2023), and even cheilocystidial morphology, a diagnostically important trait, exhibits developmental variation in both length and width, diminishing the reliability of isolated dimensional measurements (Norvell 1998b). A significant advance in generic delimitation came with the discovery of tibiiform diverticula—gland-like, aseptate, capitate structures found on the pseudorhiza and mycelium—which serve as a stable synapomorphy for the genus (Redhead and Malloch 1986; Laber 1991; Norvell 1998b).

The integration of molecular data has advanced the systematics of *Phaeocollybia*. Initial investigations employed restriction fragment length polymorphism (RFLP) analysis of the internal transcribed spacer (ITS) region of ribosomal DNA (rDNA), which proved valuable for species delimitation and revealed cryptic diversity within morphologically defined complexes (Norvell 1998a). Subsequent Sanger sequencing provided a more robust framework, enabling direct comparison of DNA sequences and the first comprehensive multigene phylogenetic reconstructions (Norvell and Exeter 2008). Sequencing of the ITS and second-largest subunit of RNA polymerase II (*rpb2*) genes from hundreds of collections, primarily from North America, refined the understanding of species boundaries and evolutionary relationships (Norvell et al. 2010, 2022). These molecular studies resolved numerous taxonomic ambiguities, leading to the synonymization of several species, including *P. californica* A.H. Sm. (= *P. rufotubulina* Norvell), *P. lilacifolia* A.H. Sm. (= *P. rifflipes* Norvell), and *P. spadicea* A.H. Sm. (= *P. tibiikauffmanii* Norvell) (Norvell et al. 2022). Furthermore, the subgeneric classification proposed by Bandala and Montoya (1994) has received molecular support, with phylogenetic analyses recovering subgen. *Phaeocollybia* and subgen. *Fibulophaeocollybia* as distinct clades (Norvell et al. 2010, 2022; Chen et al. 2026). More recently, Chen et al. (2026) conducted the first multigene phylogenetic study of *Phaeocollybia* from southwestern China, describing four new species and confirming that they belong to subgen. *Phaeocollybia*. Their analyses also revealed that *P. phaeogaleroides* Norvell falls outside the two recognized subgenera, potentially representing a distinct evolutionary lineage, a topology consistent with earlier findings (Norvell et al. 2022; Chen et al. 2026). Despite these advances, phylogenetic studies have predominantly focused on North American and Australasian taxa, while Asian species remain underrepresented in global molecular datasets, leaving their phylogenetic placement and relationships largely unresolved (Chen et al. 2026).

Field surveys were conducted in Yunnan Province and the Xizang Autonomous Region from 2024 to 2025, and specimens of *Phaeocollybia* were collected. Morphological observations and multigene phylogenetic analyses of ITS, nuclear ribosomal large subunit (nrLSU), and *rpb2* led to the discovery of two new species and one newly recorded species of *Phaeocollybia* in China, and these taxa are comprehensively described herein. Species of *Phaeocollybia* are predominantly distributed in temperate and subpolar regions (Smith 1957; Horak 1977; Norvell 1998a; Norvell and Exeter 2008). Given China’s diverse geography, climate, and vegetation conditions, more species are expected to be discovered. This study aims to advance taxonomic research on *Phaeocollybia* in China, clarify its species diversity, and elucidate its distribution patterns. Furthermore, detailed morphological comparisons and an identification key for Chinese *Phaeocollybia* species are provided.

## Materials and methods

### Specimens collected

The specimens utilized in this study were collected from Yunnan Province and the Xizang Autonomous Region of China. In the field, fresh basidiomata were photographed *in situ*, and their macroscopic morphological characteristics were recorded, including collection site, date, habitat, substrate, elevation, and geographical coordinates. All specimens were dried at 55 °C using a drying oven and subsequently preserved in self-sealing bags. The specimens are deposited in the Herbarium of Cryptogams, Kunming Institute of Botany, Chinese Academy of Sciences (HKAS), and the Herbarium of Dali University.

### Morphological studies

Macromorphology was documented from fresh basidiomata; colors were matched using the Color Hexa website (https://www.colorhexa.com/) and terminology followed Bas (1990). For micromorphology, dried material was sectioned and mounted in 5% KOH. When observations were unclear, 1% Congo Red was used as a supplementary stain to assist examination. Preparations were examined with a Nikon ECLIPSE Ni-U microscope equipped with a DS-Ri2 digital camera. For SEM studies, fragments of the lamellae of the dried material were taken, sputter-coated with gold, and analyzed using a TM4000Plus II at Dali University (Hitachi, Japan). At 1,000× magnification, 20 basidia and 50 basidiospores per collection were measured. The notation [*x*/*y*/*z*] indicates that measurements were taken from *x* basidiospores from *y* basidiomata in *z* collections. Basidiospore dimensions are presented as ‘(*a*) *b*–*n*–*c* (*d*)’, where *a* and *d* represent the minimum and maximum values of all measurements, respectively, *b*–*c* represents the range of 95% of the measured basidiospores, *n* refers to the average measurement, *Q* is the length/width ratio of basidiospores, and ‘*Q_m_*’ is the average *Q* value ± standard deviation of all basidiospores (Tang et al. 2025).

### DNA extraction, PCR amplification, and sequencing

DNA was extracted from dried specimens using the Ezup Column Fungi Genomic DNA Extraction Kit (Sangon Biotech, Shanghai, China) following the manufacturer’s protocol. For PCR amplification, the PCR mixture contained 1 µL of each primer, 1 µL of extracted DNA, 9.5 µL of ddH_2_O, and 12.5 µL of 2× EasyTaq PCR SuperMix (Sangon Biotechnology Co., Kunming, China). The ITS region was amplified using primers ITS1 and ITS4 (White et al. 1990), nrLSU using LR0R and LR5 (Vilgalys and Hester 1990), and *rpb2* using 6F and 7.1R (Liu et al. 1999). PCR was conducted using a C1000 Thermal Cycler (Bio-Rad, Beijing, China) under the following conditions: for ITS and nrLSU, initial denaturation at 94 °C for 5 min, 35 cycles of denaturation at 94 °C for 30 s, annealing at 48 °C for 30 s, extension at 72 °C for 90 s, and a final extension at 72 °C for 10 min (White et al. 1990); for *rpb2*, initial denaturation at 95 °C for 5 min, 35 cycles of denaturation at 95 °C for 60 s, annealing at 50 °C for 90 s, extension at 72 °C for 60 s, and a final extension at 72 °C for 10 min. The PCR products were sent to Sangon Biotech (Kunming, Yunnan Province, China) for sequencing.

### Sequence alignment and phylogenetic analyses

The newly generated sequences from this study were submitted to GenBank (https://www.ncbi.nlm.nih.gov/genbank/), and additional related sequences were retrieved from the NCBI (https://www.ncbi.nlm.nih.gov/) datasets, with *Galerina semilanceata* (Peck) A.H. Sm. & Singer designated as the outgroup (Norvell et al. 2022). Clusters of closely related sequences were examined for comparative purposes (see Table 1). Multiple sequence alignment was conducted using MAFFT v. 7 (Katoh and Standley 2013), followed by manual trimming and quality assessment in BioEdit v. 7 (Hall et al. 2011). Maximum likelihood (ML) analysis was subsequently conducted using RAxML-HPC2 v. 8.2.3 (Stamatakis 2014), implemented on the CIPRES platform (Miller et al. 2010), applying the GTR+G+I substitution model with 1,000 rapid bootstrap replicates. The three single-gene alignments were then concatenated into a combined dataset using Sequence Matrix (Vaidya et al. 2011).

**Table 1.** Names, voucher numbers, origin, and GenBank accession numbers of the sequences used in this study. Newly generated sequences are shown in bold. “*” following a species name indicates that the specimen is the type of that species; “–” indicates that sequence data are unavailable.

**Taxon name**	**Voucher numbers**	**Origin**	**ITS**	**LSU**	***rpb2***	**Reference**
*Phaeocollybia ammiratii*	LLN1941028-10*	USA	JN102495	–	–	Norvell et al. 2022
*P. ammiratii*	LLN2051018-01	USA	GQ165629	–	–	Norvell et al. 2022
*P. ammiratii*	OSC 155802	USA	KJ450913	–	–	Norvell et al. 2022
*P. ammiratii*	RLE2007-105	USA	GQ165624	–	–	Norvell et al. 2022
*P. attenuata*	LLN2071029-51	USA	JN102501	–	–	Norvell et al. 2022
*P. attenuata*	RLE2007-026	USA	MZ352106	–	KU574794	Norvell et al. 2022
***P. attenuata***	**HKAS 154131**	**China**	**PZ518226**	**PZ518219**	**PZ943440**	**This study**
***P. attenuata***	**HKAS 154132**	**China**	**PZ518227**	**PZ518220**	**PZ943441**	**This study**
*P. benzokauffmanii*	LLN1921120-01*	USA	GQ165636	–	–	Norvell et al. 2022
*P. benzokauffmanii*	RLE2009-15	USA	JN102502	–	KU574785	Norvell et al. 2022
*P. californica*	MICH 11607*	USA	JN102503	–	–	Norvell et al. 2022
*P. californica*	RLE2007-177	USA	GQ165647	–	KU574764	Norvell et al. 2022
*P. californica*	RLE2010-05	USA	JN102504	–	–	Norvell et al. 2022
*P. californica*	P353i	USA	EU669237	EU669287	–	Norvell et al. 2022
*P. chefensis*	RLE2009-04*	USA	MZ352102	–	KU574784	Norvell et al. 2022
*P. chefensis*	RLE2007-074	USA	MZ352097	–	KU574779	Norvell et al. 2022
*P. christinae*	MCVE 3539	Italy	JF908573	–	–	Norvell et al. 2022
*P. cidaris*	KR-M-0039954	Germany	OL771996	–	–	Norvell et al. 2022
*P. conicogregaria*	HKAS 149950*	China	PV938510	–	PV976670	Chen et al. 2026
*P. conicogregaria*	HKAS 149951	China	PV938512	–	PV976672	Chen et al. 2026
*P. dissiliens*	OSC 114217	USA	EU846271	–	–	Norvell et al. 2022
*P. dissiliens*	RLE2008-138	USA	KF219569	–	KU574767	Norvell et al. 2022
***P. dingjieensis***	HKAS 154723*	**China**	**PZ518229**	**PZ518222**	**PZ943444**	**This study**
***P. dingjieensis***	**HKAS 154721**	**China**	**PZ518230**	**PZ518223**	**PZ943443**	**This study**
***P. dingjieensis***	**HKAS 154720**	**China**	**PZ518228**	**PZ518221**	**PZ943442**	**This study**
*P. elegans*	PDD 72723	New Zealand	KY827313	–	–	Norvell et al. 2022
*P. fallax*	LLN1921007-01	USA	JN102512	–	–	Norvell et al. 2022
*P. fallax*	RLE2007-140	USA	MZ352103	–	KU574791	Norvell et al. 2022
*P. fallax*	RLE2007-159	USA	JN102516	–	KU574795	Norvell et al. 2022
*P. festiva*	WTU-F-053245	Norway	DQ494682	–	–	Norvell et al. 2022
*P. gracilis*	PDD 88665	New Zealand	KY827314	KY827262	–	Norvell et al. 2022
*P. graveolens*	PERTH 5311586	Australia	AF501567	–	–	Norvell et al. 2022
*P. gregaria*	LLN2091026cg8	USA	JN102520	–	–	Norvell et al. 2022
*P. gregaria*	RLE2008-026	USA	GQ165654	–	KU574772	Norvell et al. 2022
*P. jennyae*	SL2357	Singapore	PZ539256	–	–	Direct Submissionm
*P. jennyae*	SL1120	Singapore	PZ539252	–	–	Direct Submissionm
*P. kauffmanii*	LLN1931015-02	USA	JN102542	–	–	Norvell et al. 2022
*P. kauffmanii*	RLE2006-10	USA	KF219573	–	KU574782	Norvell et al. 2022
*P. kauffmanii*	RLE2008-027	USA	KF219572	–	KU574781	Norvell et al. 2022
*P. kauffmanii*	RLE2008-045	USA	JN102526	–	–	Norvell et al. 2022
*P. lilacifolia*	LLN1921111-06	USA	KF219576	–	–	Norvell et al. 2022
*P. lilacifolia*	LLN2071018-12	USA	GQ165657	–	KU574763	Norvell et al. 2022
*P. lugubris*	MCVE 14619	Italy	JF908574	–	–	Norvell et al. 2022
***P. lulangensis***	HKAS 154127*	**China**	**PZ518224**	**PZ518217**	**PZ943445**	**This study**
***P. lulangensis***	**HKAS 154128**	**China**	**PZ518225**	**PZ518218**	**PZ943446**	**This study**
*P. luteosquamulosa*	LLN2071029-33	USA	GQ165668	–	KU574774	Norvell et al. 2022
*P. luteosquamulosa*	LLN2071029-43	USA	GQ165667	–	–	Norvell et al. 2022
*P. megalospora*	SP 445402	Brazil	KC662116	KC662117	–	Norvell et al. 2022
*P. ochraceocana*	RLE2007-033*	USA	GQ165674	–	KU574775	Norvell et al. 2022
*P. ochraceocana*	OSC 134678	USA	GQ165672	–	–	Norvell et al. 2022
*P. ochrilata*	HKAS 148985*	China	PV938527	–	PV976687	Chen et al. 2026
*P. ochrilata*	HKAS 148987	China	PV938470	–	PV976630	Chen et al. 2026
*P. olivacea*	LLN1921015-03	USA	JN102533	–	–	Norvell et al. 2022
*P. olivacea*	RLE2007-122	USA	JN102534	–	KU574798	Norvell et al. 2022
*P. olivacea*	RLE2007-133	USA	JN102535	–	KU574799	Norvell et al. 2022
*P. olivacea*	RLE2008-050	USA	MZ352094	–	–	Norvell et al. 2022
*P. oregonensis*	LLN2001105-01	USA	GQ165681	–	–	Norvell et al. 2022
*P. pakistanica*	SWAT 15-1560*	Pakistan	KY007615	–	–	Norvell et al. 2022
*P. pakistanica*	SWAT 15-1561	Pakistan	KY007616	–	–	Norvell et al. 2022
*P. phaeogaleroides*	RLE199-064a*	USA	OM065392	–	–	Norvell et al. 2022
*P. phaeogaleroides*	RLE2009-29a	USA	MF737171	–	KU574789	Norvell et al. 2022
*P. piceae*	MICH 11629*	USA	MF737169	–	KU574762	Norvell et al. 2022
*P. pleurocystidiata*	LLN1940330-02-iso*	USA	GQ165688	–	–	Norvell et al. 2022
*P. pleurocystidiata*	RLE2008-002	USA	GQ165687	–	KU574765	Norvell et al. 2022
*P. profundirhizoidea*	HKAS 148983*	China	PV938475	–	PV976635	Chen et al. 2026
*P. profundirhizoidea*	HKAS 148984	China	PV938476	–	PV976636	Chen et al. 2026
*P. pseudofestiva*	LLN1921104-10	USA	KF219588	–	ON524835	Norvell et al. 2022
*P. pseudofestiva*	RLE2007-069	USA	KF219589	–	KU574797	Norvell et al. 2022
*P. purpureorosea*	HKAS 142478*	China	PV938535	–	PV976695	Chen et al. 2026
*P. purpureorosea*	HKAS 148982	China	PV938536	–	PV976696	Chen et al. 2026
*P. radicata*	OSC 112980	USA	EU846275	–	–	Norvell et al. 2022
*P. radicata*	RLE2008-131	USA	GQ165695	–	KU574771	Norvell et al. 2022
*P. ratticauda*	PDD 72678	New Zealand	KY827316	–	–	Norvell et al. 2022
*P. ratticauda*	PDD 72544	New Zealand	KY827315	KY827263	–	Norvell et al. 2022
*P. redheadii*	LLN2071018-17	USA	JN102544	–	–	Norvell et al. 2022
*P. redheadii*	RLE2008-038	USA	MZ352098	–	KU574783	Norvell et al. 2022
*P. scatesiae*	LLN1921015-19	USA	GQ165701	–	–	Norvell et al. 2022
*P. scatesiae*	LLN2071029-02	USA	GQ165699	–	KU574768	Norvell et al. 2022
*P. scatesiae*	RLE2007-151	USA	GQ165700	–	KU574769	Norvell et al. 2022
*P. scatesiae*	p275i	USA	EU669352	EU669395	–	Norvell et al. 2022
*P. sipei*	LLN1971023-69	USA	EU644706	–	–	Norvell et al. 2022
*P. sipei*	RLE2007-123	USA	GQ165704	–	KU574773	Norvell et al. 2022
*P. spadicea*	OSC 112482	USA	EU697252	–	–	Norvell et al. 2022
*P. spadicea*	RLE2009-18	USA	JN102550	–	KU574786	Norvell et al. 2022
*P. spadicea*	RLE2009-20	USA	JN102551	–	KU574788	Norvell et al. 2022
*P. spadicea*	P310i	USA	EU669361	EU669405	–	Norvell et al. 2022
*P. tenuis*	PDD 72672	New Zealand	KY827318	KY827264	–	Norvell et al. 2022
*Galerina semilanceata*	WTU-F-007080	USA	DQ486706	–	–	Norvell et al. 2022
*G. semilanceata*	OSC252436105	USA	PX657753	PX658108	–	Norvell et al. 2022

Bayesian inference was performed using MrBayes v. 3.2 (Ronquist et al. 2012). The concatenated dataset was partitioned by gene region (ITS, nrLSU, and *rpb2*). The best-fit substitution models for each partition were selected using MrModeltest v. 2.3 (Nylander et al. 2004) under the Akaike information criterion (AIC). Two independent runs of six Markov chain Monte Carlo (MCMC) chains each were conducted for 10,000,000 generations, with trees sampled every 1,000 generations and the first 25% discarded as burn-in (Rannala and Yang 1996). The runs were terminated automatically using the stoprule command once the average standard deviation of split frequencies fell below 0.01. A clade was considered strongly supported if it received bootstrap support (BS) ≥ 75% in the ML analysis and Bayesian posterior probability (PP) ≥ 0.90.

## Results

### Molecular phylogeny

In this study, 21 new sequences were generated from seven samples, comprising seven ITS, seven nrLSU, and seven *rpb2* sequences. All newly generated sequences have been deposited in the National Center for Biotechnology Information (NCBI) database. For phylogenetic analyses, 150 sequences were utilized, including 91 ITS, 16 nrLSU, and 43 *rpb2* sequences. These sequences were derived from 91 samples representing 42 species, with *Galerina semilanceata* (WTU-F-007080 and OSC252436105) designated as the outgroup (Norvell et al. 2022). The concatenated dataset of three loci (ITS: 1–557 bp; nrLSU: 558–1,386 bp; *rpb2*: 1,387–2,030 bp) yielded a total alignment length of 2,030 bp (including gaps).

**Figure 1.**
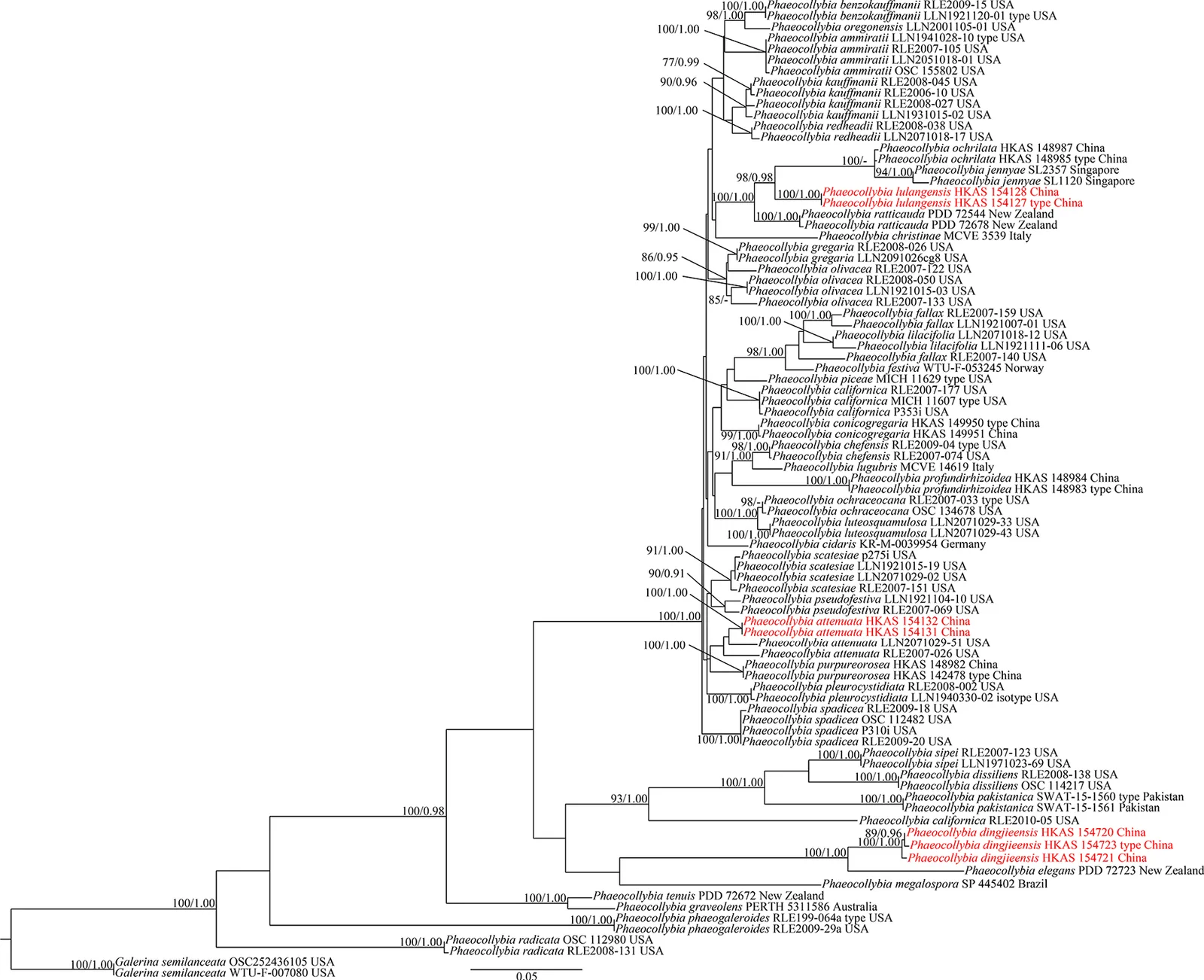
Phylogenetic relationships inferred from the concatenated sequences of ITS + LSU + *rpb*2, presented as a strict consensus tree. Support values at nodes represent maximum likelihood bootstrap proportions (≥ 75%) and Bayesian posterior probabilities (≥ 0.90). Sequences obtained in this study are indicated in red.

The phylogenetic analysis of the combined dataset using RAxML resulted in a best-scoring tree with a likelihood of −12654.703671. The alignment matrix contained 722 distinct patterns, with 52.65% undetermined or gapped. The estimated base frequencies were A = 0.266747, C = 0.195304, G = 0.235226, and T = 0.302723; substitution rates were AC = 1.047191, AG = 3.303728, AT = 1.494945, CG = 0.488742, CT = 4.604515, and GT = 1.000000; *α* = 0.233548; and tree length = 2.116969. The best-fit model for Bayesian analysis of all three single-gene datasets, determined using MrModeltest v. 2.3, was GTR+I+G [Lset nst = 6, rates = invgamma; Prset statefreqpr = dirichlet (1, 1, 1, 1)]. The ML and BI analyses generated nearly identical tree topologies with virtually no difference in statistical support. Therefore, only the ML tree is shown (Fig. 1). Multilocus phylogenetic analysis revealed that the seven *Phaeocollybia* samples formed three well-supported independent clades (Fig. 1), which are described as two new species and one newly recorded species in China. *Phaeocollybia dingjieensis* formed a strongly supported trichotomy with *P. elegans* (100% ML/1.00 PP). *Phaeocollybia lulangensis* (100% ML/–) formed a sister clade to the clade comprising *P. ochrilata* Yan C. Li, San. Z. Chen & Zhu L. Yang and *P. jennyae* (P. Karst.) Romagn. The newly recorded species *P. attenuata* (HKAS 154131 and HKAS 154132) from China formed a sister clade with *P. attenuata* (LLN2071029-51) from the USA, albeit with poor bootstrap support.

The results of the ITS molecular data comparisons of the two type specimens are shown in Table 2.

**Table 2.** BLAST results for the two new species and one known species showing the five closest taxa and their corresponding parameters.

**Scientific Name**	**Specimens**	**Sequence**	**Voucher**	**Max score**	**Total score**	**Query cove**	**Per. Ident**.	**Acc. length**	**Accession**	**Reference**
*Phaeocollybia attenuata*	HKAS 154131	ITS	HYW261	1075	1075	98%	98.84%	794	OR035525	Unpublished
			Li7515	1050	1050	95%	99.31%	579	PV938494	Chen et al. 2026
			Li7356	1038	1038	94%	99.14%	576	PV938493	Chen et al. 2026
			Li7376	1031	1031	94%	98.96%	574	PV938492	Chen et al. 2026
			iNaturalist # 191817426	1024	1024	98%	97.21%	689	PQ817069	Unpublished
*Phaeocollybia dingjieensis*	HKAS 154723	ITS	ZSY624	1014	1014	97%	99.82%	556	PV938472	Chen et al. 2026
			Li4378	902	902	97%	96.06%	560	PV938471	Chen et al. 2026
			PDD:72723	693	693	100%	88.61%	688	KY827313	Horak 2018
			Dipt15-SL2A	643	643	100%	87.25%	655	LC482596	Unpublished
			AC47A	616	616	100%	86.50%	882	KM594985	Unpublished
*Phaeocollybia lulangensis*	HKAS 154127	ITS	Li7803	966	966	96%	96.60%	691	PV938469	Chen et al. 2026
			Li7793	966	966	96%	96.60%	668	PV938461	Chen et al. 2026
			rpr-420	966	966	96%	96.60%	637	MW374195	Unpublished
			LFF358	963	963	91%	98.04%	550	PV938533	Chen et al. 2026
			Li6926	948	948	91%	97.67%	549	PV938534	Chen et al. 2026

### Taxonomy

#### Phaeocollybia dingjieensis

Taxon classificationFungiAgaricalesHymenogastraceae

R.Y. Li & S.M. Tang sp. nov.

593CC61F-9436-57BF-9D14-7B63D663F530

Fungal Names: FN 574012

**Figure 2.**
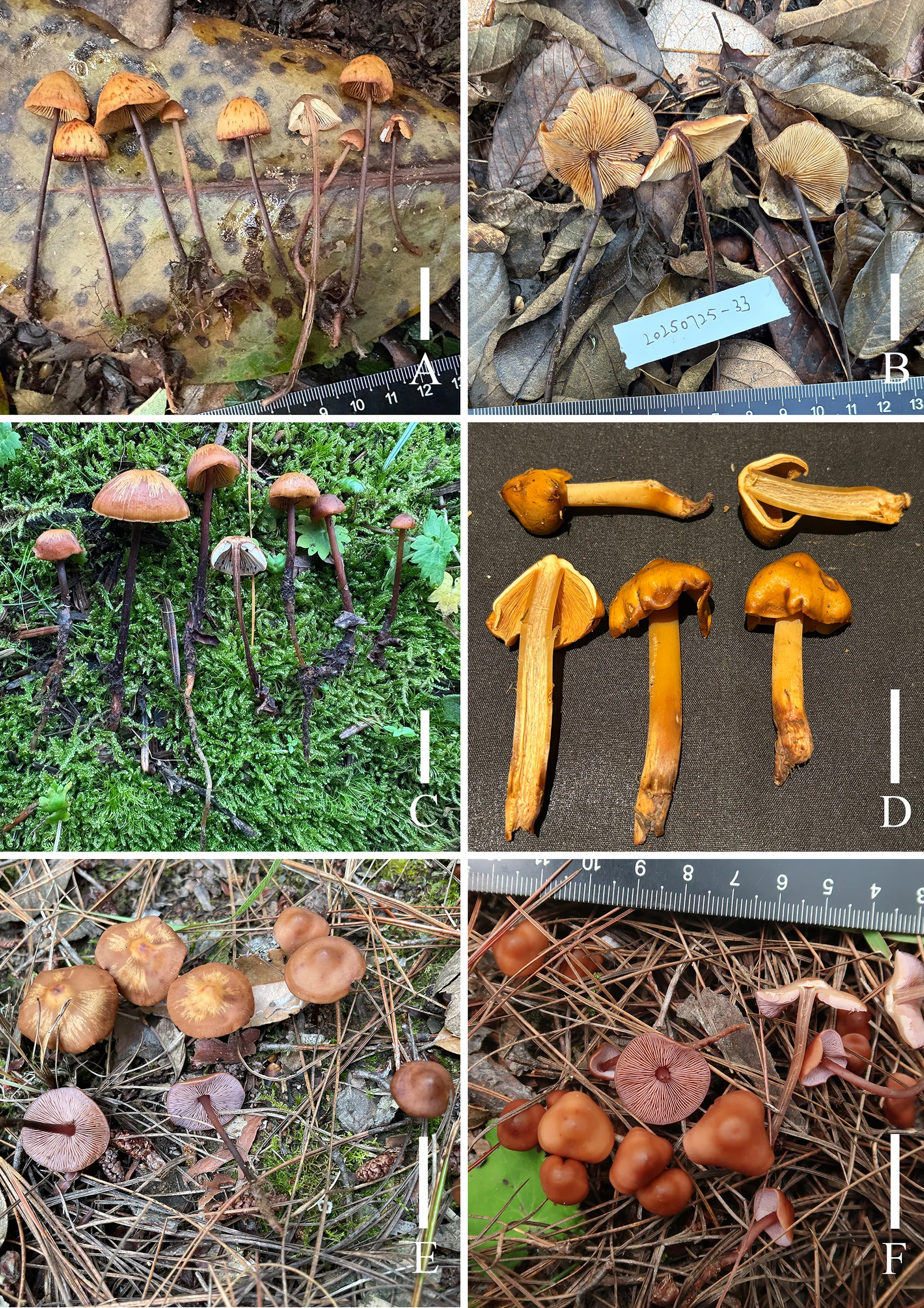
Basidiomata of *Phaeocollybia*. **A–C**. *P. dingjieensis* (**A**. HKAS 154723, holotype; **B**. HKAS 154720, paratype; **C**. HKAS 154721); **D**. *P. lulangensis* (HKAS 154127, holotype); **E, F**. *P. attenuata* (**E**. HKAS 154131; **F**. HKAS 154132). Scale bars: 2 cm.

**Figure 3.**
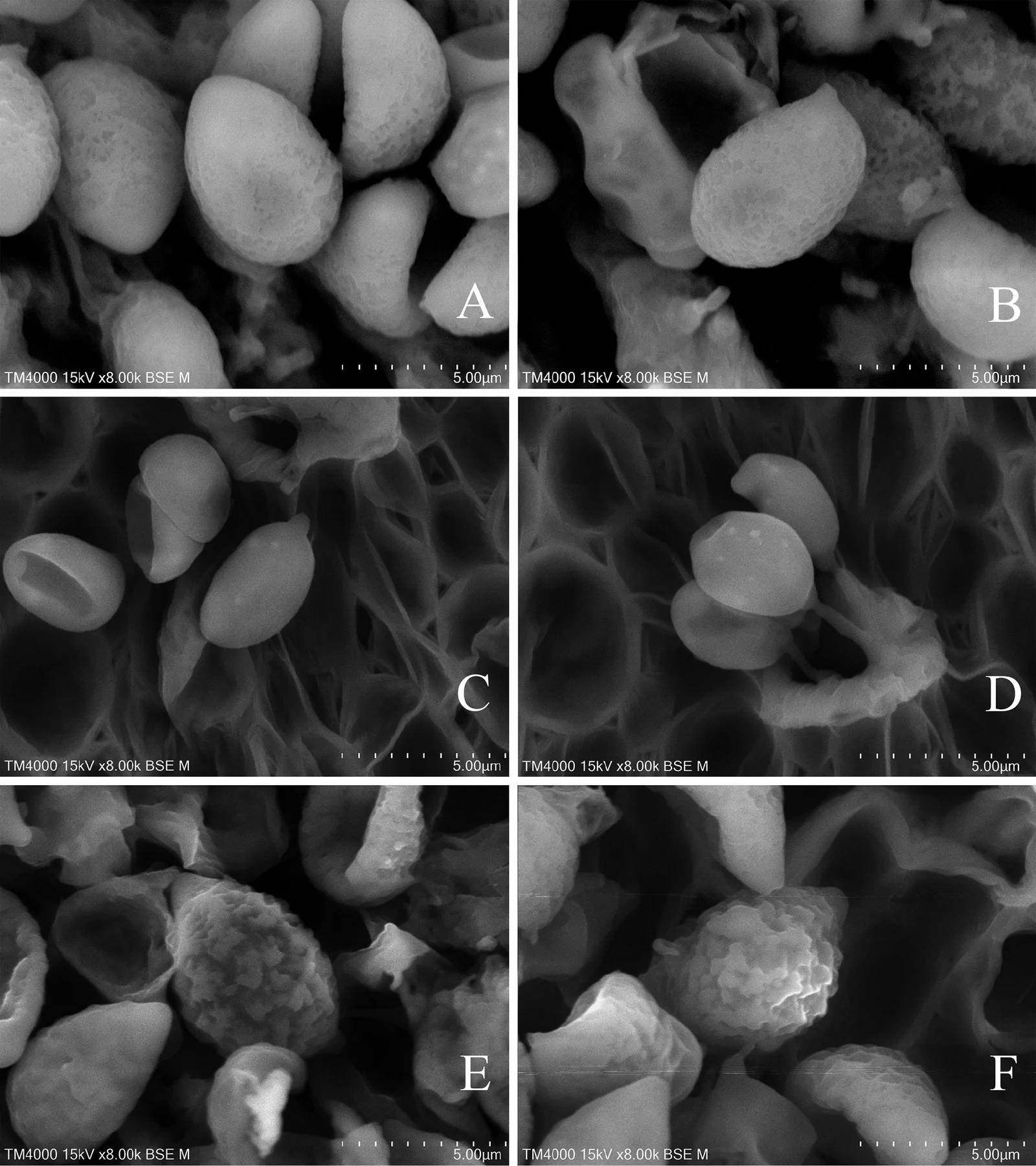
Basidiospores under electron microscopy of *Phaeocollybia*. **A, B**. *P. dingjieensis* (HKAS 154723, holotype); **C, D**. *P. lulangensis* (HKAS 154127, holotype); **E, F**. *P. attenuata* (HKAS 154131). Scale bars: 5 μm.

**Figure 4.**
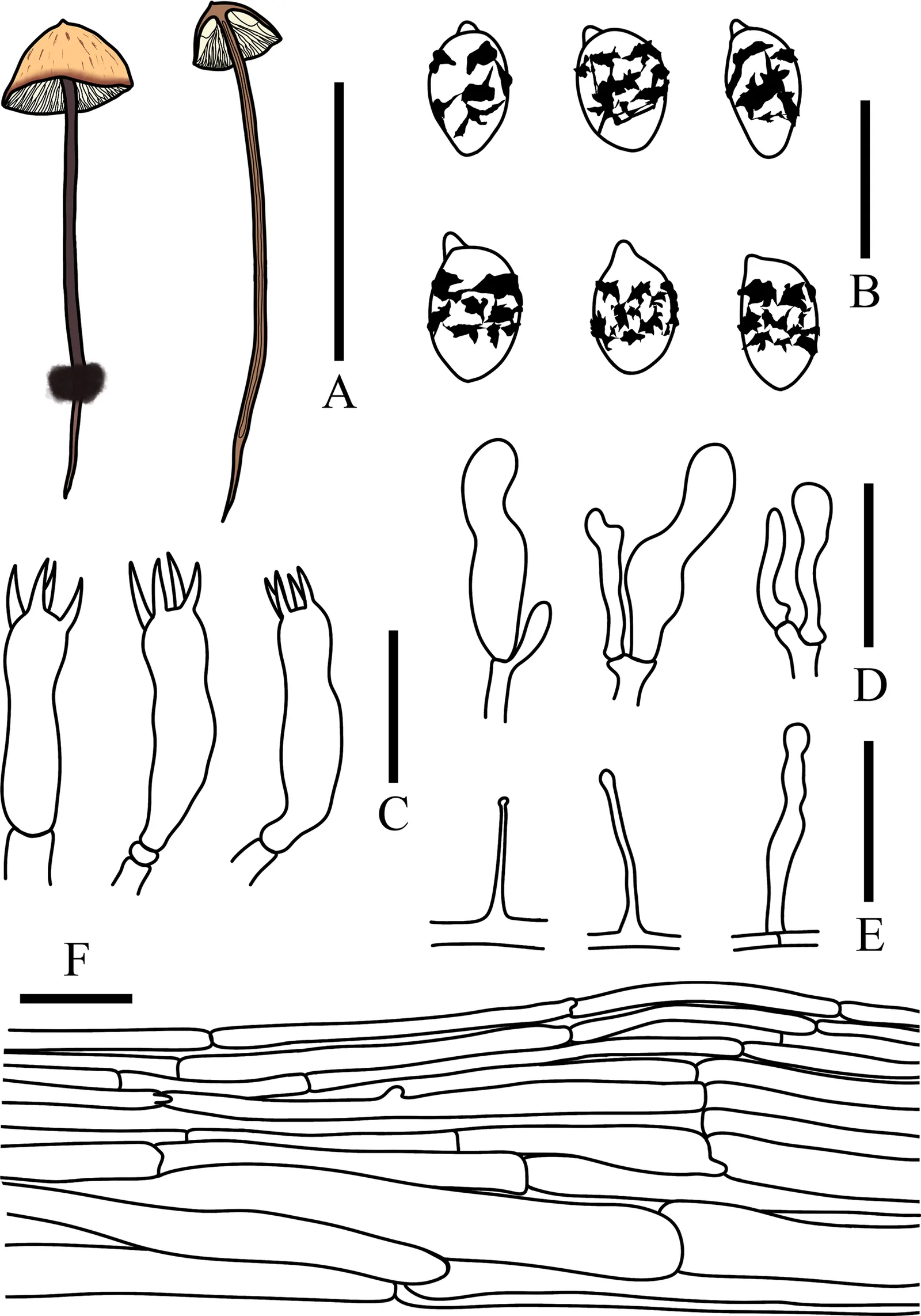
*Phaeocollybia dingjieensis* (HKAS 154723, holotype). **A**. Basidioma; **B**. Basidiospores; **C**. Basidia; **D**. Cheilocystidia; **E**. Tibiiform diverticula; **F**. Pileipellis. Scale bars: 5 cm (**A**); 10 μm (**B**); 20 μm (**C–F**).

Figs 2A–C, 3A, 3B, 4

##### Chinese name.

定结暗金钱菌 (Ding Jie An Jin Qian Jun).

##### Etymology.

The specific epithet “dingjieensis” refers to Dingjie County, Xizang Autonomous Region, China, where the holotype was collected.

##### Diagnosis.

*Phaeocollybia dingjieensis* is distinguished by a light orange pileus with dark orange vertical stripes, a long hollow stipe turning dark desaturated red at maturity, limoniform basidiospores (7.6–9.2 × 4.6–5.8 μm) with verruculose ornamentation (apex and apiculus smooth), and the absence of pleurocystidia. Clamp connections occasionally.

##### Holotype.

China • Xizang Autonomous Region, Dingjie County, Chentang Town, elev. 3,332 m, 25 July 2025, Rui-Yu Li (HKAS 154723!).

##### Description.

***Pileus*** 1.5–2.5 cm diam, paraboloid to broadly conical with acute papilla in center, margin subinvolute and subundate edge, mostly light orange (#ffce77), margins dark moderate orange (#a36c55), dark moderate orange vertical stripes over the surface, pileus context thin, 0.1–0.2 cm thick, slightly desaturated orange (#b38769). ***Lamellae*** ventricose, adnexed to narrowly adnate, moderately close together, edge undate, 3–5 irregularly arranged tiers, 2–3 mm wide, light grayish yellow (#f4eed6). ***Stipe*** central, cylindrical, hollow, 8–10 cm long, 0.2–0.3 cm diam, smooth in surface, soft orange (#edc0af) when young, becoming dark desaturated red (#55373b) at maturity, stipe context concolorous with pileus context. ***Pseudorhiza*** 1–4 cm long, hollow, rhizomorphic, cylindrical and hollow above, tapering below, soft orange (#edc0af).

***Basidiospores*** [100/2/2] (6.5–)7.6–9.2 × 4.6–5.8 μm, (*N* = 100, *Q* = 1.10–2.27, *Q_m_* = 1.63 ± 0.18, av. 8.43 ± 0.63 × 5.20 ± 0.47 μm, in face view limoniform with an apical papilla; in lateral view amygdaliform with a slight suprahilar depression; apical and apiculus smooth, the remaining surface verruculose to verrucose; brown-toned in 1% Congo Red and 5% KOH solution. ***Basidia*** 24–32 × 6–8 μm (*N* = 20), av. 28.6 ± 1.9 × 7.0 ± 0.4 μm, 2–4-spored, sterigmata 3.6–7.1 μm long, narrowly clavate, or cylindrical with median constriction, flexuose, guttulate. ***Cheilocystidia*** 14–40 × 2–9 μm (*N* = 20), av. 26.5 ± 5.7 × 6.1 ± 1.6 μm, narowly utriform, cylindrical with median constriction, or narrowly cylindrical, flexuose, occasionally branched. ***Pleurocystidia*** not observed. ***Subhymenium*** thin-walled, 5–10 μm thick, with 2–3 layers of ovoid, fusiform or globose, and 2.3–6.8 × 1.9–5.0 μm, av. 3.4 ± 1.1 × 3.3 ± 0.8 μm. ***Hymenophoral trama*** subregular, 25–55 μm wide, consisting of thin and hyaline hyphae, narrowly cylindrical hyphal elements, 3–7 μm wide. ***Pileipellis*** two-layered: upper layer ≤ 30 μm thick, a subparallel layer of thin hyphae 1–4 μm wide; lower layer ≤ 60 μm thick, of parallel hyphae 8–12 μm wide, constricted at the septa. ***Stipitipellis*** parallel aligned, thin-walled, hyphae 3–6 µm wide, sometimes constricted at the septal. ***Tibiiform diverticula*** abundant on pseudorrhizal pellis, 10–25 × 2–4 μm (*N* = 14), av. 17.1 ± 3.5 × 2.6 ± 0.5 μm, flexuose, narrowly cylindrical to narrowly conical with subcapitate apex, transparent and colorless, thin-walled. ***Clamp connections*** occasionally present in pileipellis.

##### Habitat.

Solitary or scattered in broad-leaved forests.

##### Additional specimens examined.

China • Xizang Autonomous Region, Yadong County, elev. 3,130 m, 24 July 2025, Song-Ming Tang, (HKAS 154720), paratype; China • ibid., Dingjie County, Chentang Town, elev. 3,332 m, 25 July 2025, Rui-Yu Li, (HKAS 154721).

##### Notes.

*Phaeocollybia dingjieensis* and *P. amygdalospora* Bandala & E. Horak are similar in having a pileus with orange to yellowish-brown tones and a brownish stipe. However, *P. amygdalospora* differs by having a smaller pileus (up to 15 mm vs. 15–25 mm in *P. dingjieensis*), which becomes flattened with age and exhibits purplish tones, and a shorter stipe (up to 60 mm vs. 80–100 mm in *P. dingjieensis*). Microscopically, the basidia of *P. amygdalospora* are typically claviform, whereas those of *P. dingjieensis* often exhibit a median constriction and flexuosity. Additionally, *P. amygdalospora* completely lacks clamp connections in all tissues (Bandala et al. 1996), while *P. dingjieensis* has occasional clamp connections. These differences support the recognition of *P. dingjieensis* as a distinct species from *P. amygdalospora*.

In the phylogenetic tree (Fig. 1), *Phaeocollybia dingjieensis* forms a sister clade to *P. elegans* E. Horak. However, *P. elegans*, with its type locality in New Zealand, differs from *P. dingjieensis* by having a smaller pileus (0.8–2.0 cm diam.) that is broadly conical, colored very dark orange at the center and fading to bright orange toward the margin, with a peeling pileipellis at maturity, and lacking both the light orange coloration with dark moderate orange vertical stripes and the acute central papilla characteristic of *P. dingjieensis*. Furthermore, *P. elegans* possesses a shorter stipe (4–6 cm vs. 8–10 cm) of a uniform very dark orange color. Microscopically, *P. elegans* produces oblong to limoniform basidiospores that are substantially smaller than those of *P. dingjieensis* (5 × 2.5 μm vs. averaging 8.4 × 5.2 μm); the latter species is further distinguished by verruculose to verrucose spore ornamentation, except at the apex and apiculus. The cheilocystidia of *P. elegans* measure 12–25 μm and are characterized by apices that are either sharply pointed (mucronate) or swollen into a spherical or subcapitate shape, whereas those of *P. dingjieensis* are longer (14–40 μm), narrowly utriform to cylindrical, sometimes with a median constriction, and occasionally branched. Clamp connections, occasionally observed in the pileipellis of *P. dingjieensis*, have not been reported for *P. elegans*. The ITS sequence difference between *P. dingjieensis* (HKAS 154723, holotype) and *P. elegans* (PDD 72723) was 10.03% (56/558, including 11 gaps).

#### Phaeocollybia lulangensis

Taxon classificationFungiAgaricalesHymenogastraceae

R.Y. Li & S.M. Tang sp. nov.

9A86FA66-8FFD-5077-965E-E8A72892272D

Fungal Names: FN 574013

Figs 2D, 3C, 3D, 5

##### Chinese name.

鲁朗暗金钱菌 (Lu Lang An Jin Qian Jun).

**Figure 5.**
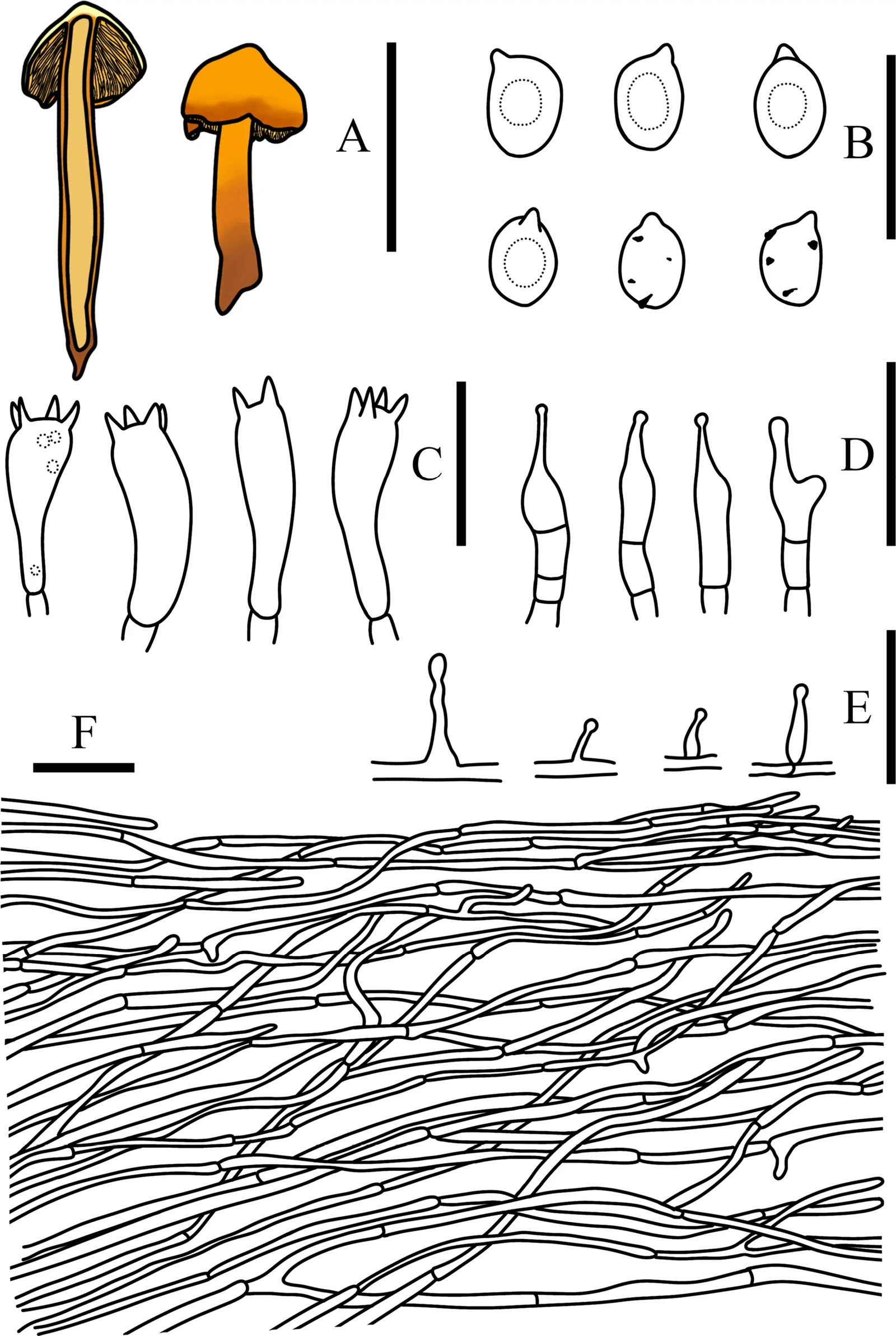
*Phaeocollybia lulangensis* (HKAS 154127, holotype). **A**. Basidioma; **B**. Basidiospores; **C**. Basidia; **D**. Cheilocystidia; **E**. Tibiiform diverticula; **F**. Pileipellis. Scale bars: 5 cm (**A**); 10 μm (**B**); 20 μm (**C–F**).

##### Etymology.

The epithet “lulangensis” refers to Lulang Town, Linzhi City, Xizang Autonomous Region, China, where the type specimen was collected.

##### Holotype.

China • Xizang Autonomous Region, Linzhi City, Lulang Town, elev. 3,419 m, 18 July 2025, Rui-Yu Li, (HKAS 154127!).

##### Diagnosis.

*Phaeocollybia lulangensis* is distinguished by a pure orange to strong orange pileus with an oily-subwaxy luster, a hollow stipe with a root-like pseudorhiza, limoniform basidiospores (5.3–6.3 × 3.8–4.6 μm) with a distinct suprahilar depression, and the absence of pleurocystidia. Clamp connections rare.

##### Description.

***Pileus*** 2.5–4.0 cm diam, broadly conical to conical with subumbonate in center, margin inflexed and even edge, often wavy, surface smooth, mostly pure orange (#fda200), deepening to strong orange (#c16728) at the center and toward the margin, with an oily, subwaxy luster, pileus context thick, 0.1–0.3 cm wide, pale yellow (#fffa9e). ***Lamellae*** arcuate, adnexed, moderately close together, edge even or entire, 3–4 irregularly arranged tiers, 1–2 mm wide, vivid orange (#ffa627). ***Stipe*** central, cylindrical, hollow, 5–8 cm long, including pseudorhiza, 0.8–1.0 cm diam, smooth in surface, subwaxy luster, pure orange (#f69900), darkening toward the base. ***Pseudorhiza*** is 1–2 cm long, root-like, somewhat hollow in the upper part, with a base that tapers abruptly and becomes solid.

***Basidiospores*** [84/2/2] 5.3–6.3 × 3.8–4.6 μm, (*N* = 84, *Q* = 1.09–1.67, *Q_m_* = 1.40 ± 0.12, av. 5.83 ± 0.49 × 4.17 ± 0.36 μm, in face view limoniform with an apical papilla; in lateral view with a profile dominated by a suprahilar depression, rarely verruculose on the smooth; brown-toned in 1% Congo Red and 5% KOH solution. ***Basidia*** 19–33 × 6–9 μm (*N* = 20), av. 26.0 ± 3.7 × 7.5 ± 0.7 μm, mostly 4-spored, rarely 2-spored, sterigmata 3.2–4.9 μm long, sometimes with basal clamp connections, cylindrical to clavate, guttulate. ***Cheilocystidia*** 12–25 × 2–5 μm (*N* = 20), av. 27.2 ± 5.7 × 4.3 ± 0.6 μm, intermixed with basidia, narowly cylindrical, lageniform or flexuose, most have a long, rostrate apex, occasionally septate. ***Pleurocystidia*** not observed. ***Subhymenium*** thin-walled, 5–8 μm thick, with 2–3 layers of ovoid, fusiform or globose, and 1.8–5.1 × 1.1–6.6 μm, av. 3.1 ± 0.8 × 3.3 ± 1.2 μm. ***Hymenophoral trama*** regular, 45–105 μm wide, consisting of thin and hyaline hyphae, narrowly cylindrical hyphal elements, 25–40 × 4–8 μm wide. ***Pileipellis*** 90–150 μm thick, with a superficial layer of parallel, repent hyphae overlying an intricate trichoderm; the hyphae are slender, loosely arranged, 1.5–2.5 μm wide. ***Stipitipellis*** parallel aligned, thin-walled, hyphae 4–12 µm wide, sometimes constricted at the septal areas. ***Tibiiform diverticula*** abundant on pseudorrhizal pellis, 3–14 × 1–3 μm (*N* = 20), av. 5.8 ± 2.8 × 1.3 ± 0.4 μm, flexuose, narrowly cylindrical to narrowly conical with subcapitate apex, transparent and colorless, thin-walled. ***Clamp connections*** absent in all tissues examined.

##### Habitat.

Solitary or scattered in broad-leaved forests.

##### Additional specimens examined.

China • Xizang Autonomous Region, Mi Lin City, elev. 3,074 m, 18 July 2025, Song-Ming Tang, (HKAS 154128), paratype.

##### Notes.

*Phaeocollybia lulangensis* is morphologically similar to *P. chefensis* Norvell & Exeter, particularly in having a broadly conical pileus with a subumbonate center and overall orange coloration. However, *P. chefensis* is currently known from the Oregon coast and Coast Range in the USA; the two species can be distinguished by the following characters: the pileus of *P. chefensis* exhibits a distinctive uniform copper metallic sheen when dry, has larger basidiospores (av. 8.9 × 5.6 μm vs. 5.8 × 4.2 μm) that are consistently verruculose to verrucose, and has tibiiform cheilocystidia with thick-walled, refractive necks and capitula (Norvell et al. 2022).

In the phylogenetic tree (Fig. 1), *P. lulangensis* forms a sister clade to *P. ratticauda* E. Horak. However, *P. ratticauda* is known from New Zealand and has a dry, reddish-brown to yellowish-brown pileus, pale violet lamellae, and heavily verrucose basidiospores, while the spore surface of *P. lulangensis* is smooth to rarely verruculose (Horak 1977). The ITS sequence difference between *P. lulangensis* (HKAS 154127, holotype) and *P. ratticauda* (PDD 72544) was 6.14% (34/553, including three gaps).

#### Phaeocollybia attenuata

Taxon classificationFungiAgaricalesHymenogastraceae

(A.H. Sm.) Singer, Lilloa 22: 567 (1951) [1949]

1973A0D3-CF74-509C-981D-F674F7ADDA64

Fungal Names: FN 302677

**Figure 6.**
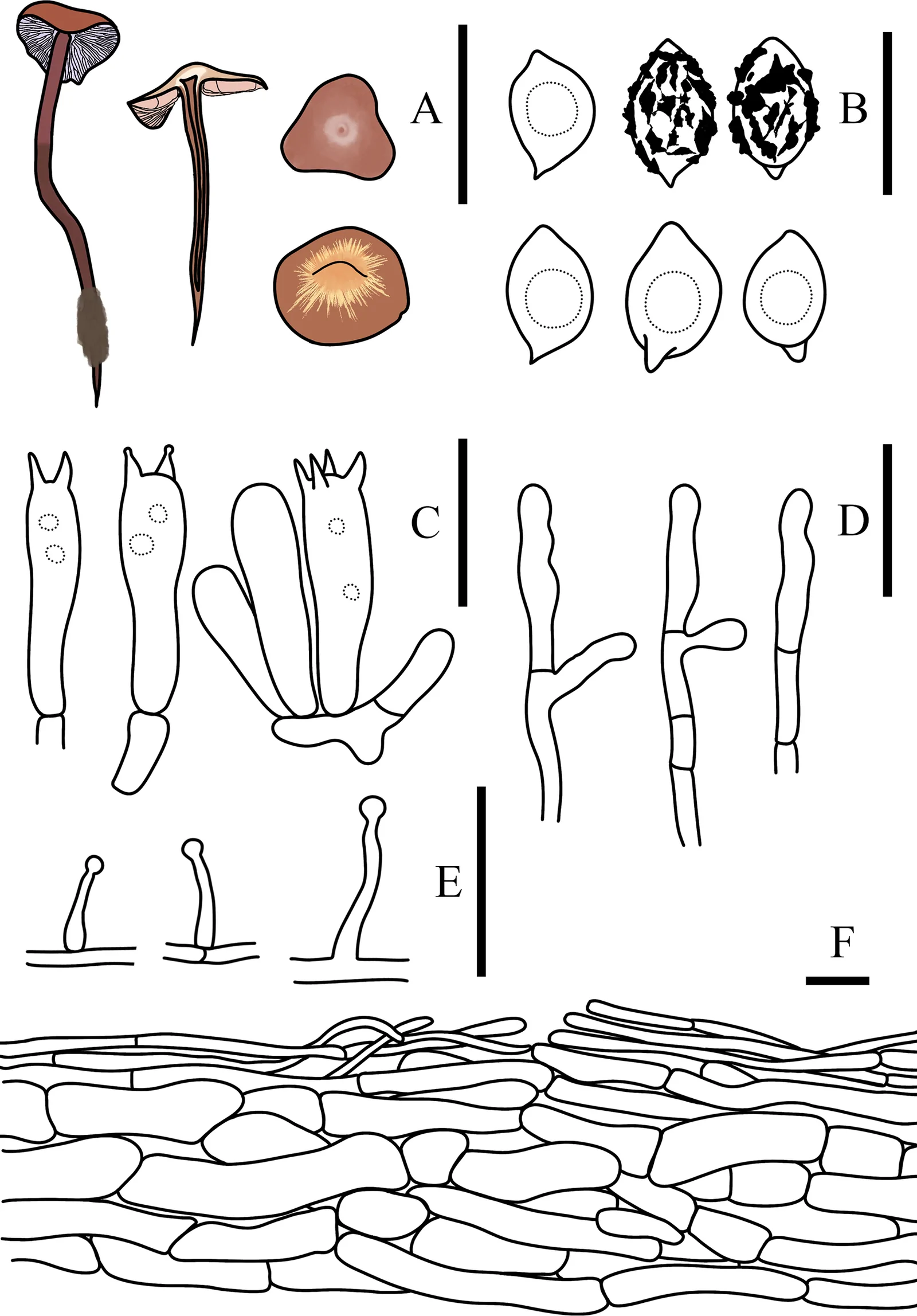
*Phaeocollybia attenuata* (HKAS 154131). **A**. Basidioma; **B**. Basidiospores; **C**. Basidia; **D**. Cheilocystidia; **E**. Tibiiform diverticula; **F**. Pileipellis. Scale bars: 2 cm (**A**); 10 μm (**B**); 20 μm (**C–F**).

Figs 2E, 2F, 3E, 3F, 6

##### Chinese name.

渐尖暗金钱菌 (Jian jian An Jin Qian Jun).

##### Description.

***Pileus*** 1.0–2.5 cm diam, convex to hemispherical with subumbonate in center, seen from above, sometimes appearing as a rounded triangle, margin straight to slightly inflexed and even edge; surface smooth, overall moderate orange (#c57753), sometimes pale orange at the center, often with abundant, radially arranged pale orange (#ffe1ae) streaks; streaks shorter near the margin; pileus context 2 mm at the center, pale orange (#ffefe1). ***Lamellae*** ventricose, nearly free, even to slighly undate edges, 3–5 irregularly arranged tiers, 1–2 mm wide, close, light grayish red (#dabdc2) when young, deepening to grayish red (#d7aba5) at maturity. ***Stipe*** central, cylindrical, hollow, 4–5 cm long, including pseudorhiza, 2–3 mm diam, smooth in surface, mostly desaturated dark red (#996a6a) on the upper portion, which gradually darkens into a dark grayish pink (#504247) on the lower half. ***Pseudorhiza*** 2–3 cm long, root-like, cylindrical and hollow in the upper part, tapering and becoming solid towards the base.

***Basidiospores*** [96/2/2] 7.9–9.8(–11.0) × (4.3–)5.3–6.5(–7.5) μm, (*N* = 96, *Q* = 1.11–1.8, *Q_m_* = 1.51 ± 0.15, av. 8.80 ± 0.73 × 5.87 ± 0.55 μm, limoniform with apical papilla, apical and apiculus smooth, the remaining surface verruculose to verrucose, brown-toned in 1% Congo Red and 5% KOH solution. ***Basidia*** 24–33 × 7–8 μm (*N* = 20), av. 28.1 ± 2.2 × 7.4 ± 0.4 μm, mostly 4-spored, rarely 2-spored, sterigmata 3.1–5.0 μm long, cylindrical to clavate, occasionally curved, guttulate. ***Cheilocystidia*** 17–38 × 3–6 μm (*N* = 20), av. 27.2 ± 5.7 × 4.3 ± 0.6 μm, intermixed with basidia, narowly cylindrical, narrowly clavate or flexuose, frequently septate. ***Pleurocystidia*** not observed. ***Subhymenium*** thin-walled, 5–10 μm thick, with 2–3 layers of ovoid, fusiform to narrowly cylindrical, and 2.3–10.7 × 2.7–7.7 μm, av. 4.5 ± 1.8 × 4.4 ± 1.4 μm. ***Hymenophoral trama*** regular, 25–45 μm wide, consisting of thin and hyaline hyphae, narrowly cylindrical hyphal elements, 20–70 × 6–11 μm. ***Pileipellis*** two-layered: upper layer ≤ 20 μm thick, of thin, parallel hyphae 3–7 μm wide; lower layer ≥ 60 μm thick, of coarse, parallel hyphae 15–25 μm wide. ***Stipitipellis*** parallel aligned, thin-walled, hyphae 8–20 µm wide, sometimes constricted at the septal areas. ***Tibiiform diverticula*** abundant on pseudorrhizal pellis, 5–17 × 1–3 μm (*N* = 20), av. 11.4 ± 2.9 × 1.9 ± 0.3 μm, narrowly cylindrical to narrowly conical with capitate apex, transparent and colorless, thin-walled. ***Clamp connections*** absent in all tissues.

##### Additional specimens examined.

China • Yunnan Province, Dali City, Jianchuan County, elev. 2,441 m, 7 September 2024, Rui-Yu Li, (HKAS 154131); • ibid., Song-Ming Tang, (HKAS 154132).

##### Notes.

*Phaeocollybia attenuata* is reported here for the first time from China (Smith 1937; Wei et al. 2012). In the phylogenetic tree (Fig. 1), *P. attenuata* (HKAS 154131) forms a sister clade to *P. attenuata* (LLN2071029-51) and *P. attenuata* (RLE2007-026). The Chinese material closely matches the holotype description by Smith in essential diagnostic features, including the presence of a long tapering pseudorhiza, a hollow cartilaginous stipe, the absence of clamp connections (except for very rare and scattered ones in the stipitipellis), and undifferentiated cystidia (Smith 1937). However, the Chinese collections exhibit some morphological variation and provide refined microcharacters not detailed in the protologue. The pileus in Chinese specimens is moderate orange with conspicuous pale orange radial streaks, a feature not mentioned in the original description. The basidiospores are slightly larger (av. 8.80 ± 0.73 × 5.87 ± 0.55 μm) and limoniform with a distinct apical papilla and verrucose ornamentation, whereas Smith described them as smaller (7–8 × 4–4.5 μm) and almond-shaped. Furthermore, detailed microscopic examination of the Chinese material reveals abundant tibiiform diverticula on the pseudorhizal pellis, a characteristic structure of the genus not noted in the original description. Despite these differences, the comprehensive analysis of morphological characteristics and phylogeny supports the identification of the Chinese collections as *P. attenuata*, indicating that the known distribution range of this species extends into East Asia, in addition to its type locality in the USA.

## Discussion

To date, 19 species of *Phaeocollybia* have been documented in China, including the three reported in this study. Of these, *P. conicogregaria* Yan C. Li, San. Z. Chen & Zhu L. Yang, *P. ochrilata*, *P. profundirhizoidea* Yan C. Li, San. Z. Chen & Zhu L. Yang, *P. purpurea* T.Z. Wei, S.Z. Fu, P.P. Qu & Y.J. Yao, *P. purpureorosea* Yan C. Li, San. Z. Chen & Zhu L. Yang, *P. similis* (Bres.) Singer, and *P. sparsilamellae* P.G. Liu were originally described with type localities in China (Smith 1957; Liu 1995; Wei et al. 2010; Chen et al. 2026). The majority of these species have been collected from subtropical and warm-temperate broad-leaved and mixed forests in southern China, such as those in Yunnan, Sichuan, Fujian, and Guangdong (Bi 1993; Wei et al. 2012).

Globally, species of *Phaeocollybia* are predominantly distributed in temperate regions of the Northern Hemisphere, with major diversity hotspots occurring in the Pacific Northwest of North America (Washington and Oregon, USA) and in subtropical to tropical montane regions of Asia and the Neotropics, notably southwestern China, Mexico, and Colombia (Smith 1957; Horak 1977; Horak and Halling 1991; Norvell 1998a, 1998b, 2000; Norvell and Exeter 2007; Norvell et al. 2022). Despite their transoceanic separation and occurrence across both temperate and tropical latitudes, these regions share significant ecological and geological parallels that appear to have promoted elevated *Phaeocollybia* species richness. All are characterized by perhumid forest ecosystems with persistently high atmospheric humidity and substantial annual precipitation. These include the temperate rainforests of the Pacific Northwest (Norvell 1998a), the tropical montane cloud forests of the Colombian and Mexican highlands (Horak and Halling 1991), and the monsoon-influenced subtropical broad-leaved and mixed forests of southwestern China, all of which are characterized by persistent moisture and high annual rainfall. This pattern aligns with the known ecological preference of *Phaeocollybia* for mesic environments, where its requirement for constant humidity is well met (Norvell 1998b).

China has a vast territory, diverse climates, and various forest types and is one of the countries with the greatest fungal diversity in the world (Dong et al. 2024, 2025; Wang et al. 2026), with extensive forests providing favorable habitats for *Phaeocollybia*. However, only 15 species have been reported from China to date. In contrast, Norvell and Exeter (2008) documented 25 species from the temperate coastal regions of the Pacific Northwest alone. This significant disparity indicates that the taxonomic diversity, biogeographic distribution patterns, and overall mycological resource status of *Phaeocollybia* in China remain substantially understudied, with numerous additional taxa presumably awaiting discovery and formal taxonomic description.

A closer examination of the distributional data reveals that all Chinese *Phaeocollybia* records are confined to regions south of the Yangtze River, spanning six provincial-level areas—Yunnan, Sichuan, Xizang, Fujian, Guangdong, and Hainan—with no records from the vast temperate and boreal forests of northern or northeastern China (Wei et al. 2012). This pattern is unlikely to be attributed solely to climatic constraints, as suitable mesic forest habitats occur at higher latitudes in China. Instead, it may reflect historical undersampling in northern regions. Notably, southwestern China stands out as the clear center of diversity within the country, harboring the two newly described species in this study as well as several previously reported taxa (Liu 1995; Chen et al. 2026). This concentration in southwestern China echoes the pattern observed in other ectomycorrhizal agaric genera and reinforces the region’s status as a priority area for future taxonomic exploration. Given that even well-studied regions such as the Pacific Northwest have yielded new species in recent decades (Norvell et al. 2022) and that China’s southwestern forests remain comparatively underexplored mycologically, the actual species richness of *Phaeocollybia* in China is likely substantially underestimated. Future efforts integrating extensive field surveys across undersampled provinces with molecular phylogenetic analyses are needed to clarify the true diversity and distribution of this ecologically significant genus in China.

In most studies on *Phaeocollybia*, species delimitation has been primarily based on morphological characters, with molecular analyses conducted by only a few researchers (Norvell 1998a, 1998b, 2000, 2002; Norvell and Redhead 2000; Norvell et al. 2022). However, sequence data from type specimens are still lacking for many species. In the present study, phylogenetic analyses based on combined ITS, nrLSU, and *rpb2* data placed the Chinese collections of *Phaeocollybia attenuata* within the clade containing *P. attenuata* from the USA. To further quantify the genetic divergence among these collections, their ITS sequences were compared, as this region serves as the primary fungal barcode. The ITS sequence differences between the Chinese collection (HKAS 154131) and *P. attenuata* (LLN2071029-51) and *P. attenuata* (RLE2007-026) were 2.69% (16/593, including six gaps) and 4.26% (25/586, including three gaps), respectively. Based on its position in the ML tree and morphological comparison with the protologue, and given the current absence of ex-type sequences for *P. attenuata*, the Chinese collection is tentatively regarded as a new record for China. Future studies should prioritize species lacking ex-type sequences; the epitypification of such taxa, supported by molecular data derived from epitype specimens, is essential to stabilize their nomenclature and resolve the prevailing taxonomic uncertainties.

### Key to the 15 species of *Phaeocollybia* reported from China

**Table:** 

1	Pileus with distinct purple tones (dark violet, grayish purple, or brownish purple when young)	**2**
–	Pileus without purple tones, brown, yellowish-brown, rust-brown, olive, or orange	**3**
2	Basidiospores 3.5–5.0 × 3.0–4.0 μm, clamp connections present; lamellae pale violet-gray when young	***P. purpurea***
–	Basidiospores larger (8–9.5 × 4.5–6.5 μm), clamp connections present; lamellae very sparse, pileus not viscid, brown to dark brown with purplish tint	***P. sparsilamellae***
3	Pileus glutinous to viscid when moist, hygrophanous, often fading	**4**
–	Pileus dry or only slightly viscid, not distinctly hygrophanous	**10**
4	Lamellae initially grayish-blue, plumbeous-violet, or pale violet-gray, becoming rust-brown at maturity	**5**
–	Lamellae consistently yellowish-brown, ochraceous-brown, or rust-brown, without bluish tones	**6**
5	Spores with an apical germ pore; lamellae edges serrate, eroded	***P. fallax***
–	Spores without a germ pore; lamellae edges even; pileus very light orange with dark moderate orange vertical stripes	***P. dingjieensis***
6	Pileus distinctly orange (pure orange to vivid orange or moderate orange)	**7**
–	Pileus brown, yellowish-brown, rust-brown, or olive	**8**
7	Pileus pure orange; spores 5.3–6.3 × 3.8–4.6 μm, surface smooth to rarely verruculose	***P. lulangensis***
–	Pileus moderate orange with pale orange radial streaks; spores 7.9–9.8 × 5.3–6.5 μm, distinctly verrucose	***P. attenuata***
8	Pileus with olive tones, fading to greenish; spores 7–9 × 4.5–5.5 μm, with apical germ pore	***P. fallax***
–	Pileus without greenish tones, brown to rust-brown; spores without germ pore	**9**
9	Pileus glutinous (gelatinous when wet); lamellae initially grayish-blue to plumbeous, becoming rusty-brown; spores with apical germ pore	***P. fallax***
–	Pileus viscid but not gelatinous; lamellae consistently lacking bluish-gray tones throughout development; spores without germ pore	***P. lugubris***
10	Pileus small (< 1.5 cm), campanulate to conic, margin striate	**11**
–	Pileus medium to large (> 2 cm), margin not striate or only faintly so	**12**
11	Spores 6–7 × 3.5–4 μm, rugulose-roughened; cuticular hyphae with abundant clamp connections	***P. minuta***
–	Spores 9–10 × 5–6.5 μm, limoniform; cuticular hyphae without clamp connections	***P. coniuncta***
12	Stipe with well-developed pseudorhiza, often longer than the epigeous portion	**13**
–	Stipe without pseudorhiza or pseudorhiza indistinct	**14**
13	Spores wider (10–11 × 6.5–8 μm); cheilocystidia capitate	***P. columbiana***
–	Spores narrower (8.5–11 × 4.5–5 μm), limoniform to amygdaliform; cystidia absent	***P. christinae***
14	Pileus up to 7 cm broad, subsquamulose; spores 6–8 × 4–5 μm, ovoid	***P. intermedia***
–	Pileus smaller (< 3.5 cm), not subsquamulose; spores larger or with apical beak	**15**
15	Spores 8–10 × 6–7 μm, broadly ovoid, distinctly beaked; cheilocystidia subgelatinous	***P. similis***
–	Spores 6.5–8 × 4–4.8 μm, amygdaliform, with smooth suprahilar plage; cheilocystidia flexuous	***P. cidaris***

## Supplementary Material

XML Treatment for Phaeocollybia dingjieensis

XML Treatment for Phaeocollybia lulangensis

XML Treatment for Phaeocollybia attenuata

## Additional information

### Artificial Intelligence (AI) use

The authors accept full responsibility for the content of the manuscript, including the disclosure of any use of AI.

No AI tools were used in the preparation of this manuscript.

### Author ORCIDs

R.-Y. Li https://orcid.org/0009-0008-2136-9460

D.-F. Zhao https://orcid.org/0009-0000-4251-3988

Y.-T. Li https://orcid.org/0009-0006-7153-751X

Y. Luo https://orcid.org/0009-0001-0244-1462

H.-B. Song https://orcid.org/0000-0002-7440-5444

H.-W. Shen https://orcid.org/0000-0003-2508-1970

Z.-L. Luo https://orcid.org/0000-0001-7307-4885

S.-M. Tang https://orcid.org/0000-0003-0665-6900

### Data availability

All of the data that support the findings of this study are available in the main text.

## References

[B1] Bandala VM, Montoya L (1994) Further investigations on *Phaeocollybia* with notes on infrageneric classification. Mycotaxon 52(2): 397–422.

[B2] Bandala VM, Montoya L, Guzmán G, Horak E (1996) Four new species of *Phaeocollybia*. Mycological Research 100(2): 239–243. 10.1016/S0953-7562(96)80130-4

[B3] Bas C [Ed.] (1990) Flora Agaricina Neerlandica (Vol. 2). CRC Press.

[B4] Bi ZJ (1993) The macrofungi flora of China’s Guangdong Province. Chinese University Press.

[B5] Chen SZ, Tan YH, Huang TW, Liang DY, Chen JG, Yang ZL, Li YC (2026) The genus *Phaeocollybia* from southwestern China based on morphological and molecular evidence. Mycologia 118(4): 789–806. 10.1080/00275514.2026.2626246 41945685

[B6] Coimbra VR, Gibertoni TB, Wartchow F (2012) *Phaeocollybia nigripes* (*Agaricomycetes*), a new species from Brazil. Mycotaxon 120(1): 171–179. 10.5248/120.171

[B7] Dong JH, Li Q, Yuan Q, Luo YX, Zhang XC, Dai YF, Zhou Q, Liu XF, Deng YL, Zhou HM, Muhammad A, Zhao CL (2024) Species diversity, taxonomy, molecular systematics and divergence time of wood-inhabiting fungi in Yunnan-Guizhou Plateau, Asia. Mycosphere 15(1): 1110–1293. 10.5943/mycosphere/15/1/10

[B8] Dong JH, Chen ML, Chen M, Li Q, Zhu YJ, Zhang XC, Zhou CQ, Li W, Muhammad A, Zhou HM, Jabeen S, Zhao CL (2025) Notes, outline, taxonomy and phylogeny of wood-inhabiting *Agaricales*. Mycosphere 16(1): 2599–2711. 10.5943/mycosphere/16/1/16

[B9] Fries EM ([1836–]1938) Epicrisis systematis mycologici, synopsis hymenomycetum. Uppsala: Typographia Academia.

[B10] Gams W (1994) Report of the committee for fungi: 4. Taxon 265–267. 10.2307/1222886

[B11] Hall T, Biosciences I, Carlsbad CJGBB (2011) BioEdit: an important software for molecular biology. GERF Bull Biosci 2(1): 60–61.

[B12] Heim R (1931) Le genre *Inocybe*. Encyclopédie Mycologique 1: 1–429.

[B13] Horak E (1977) Further additions towards a monograph of *Phaeocollybia*. Sydowia 29: 28–70.

[B14] Horak E (1980) Indian *Boletales* and *Agaricales*. Revisions and new taxa. Sydowia 33: 88–110.

[B15] Horak E (2018) *Agaricales (Basidiomycota)* of New Zealand, 2: Brown Spored Genera Pp *Crepidotus*, *Flammulaster*, *Inocybe*, *Phaeocollybia*, *Phaeomarasmius*, *Pleuroflammula*, *Pyrrhoglossum*, *Simocybe*, *Tubaria* and *Tympanella*. Fungal Diversity Press.

[B16] Horak E, Halling RE (1991) New records of *Phaeocollybia* from Colombia. Mycologia 83(4): 464–472. 10.1080/00275514.1991.12026036

[B17] Hyde KD, Amuhenage TB, Apurillo CCS, Asghari R, Aumentado HD, Bahkali AH, Bera I, Bhunjun CS, Calabon MS, Chandrasiri S, Chethana KWT, Doilom M, Dong W, Fallahi M, Gajanayake AJ, Gomdola D, Gunarathne A, Hongsanan S, Huanraluek N, Jayawardena RS, Kapov SA, Khyaju S, Le L, Li CJY, Li QR, Li YX, Lin CG, Linn MM, Liu JK, Liu NG, Luangharn T, Madagammana AD, Manawasinghe IS, Marasinghe DS, McKenzie EHC, Meakala N, Meng QF, Mukhopadhyay S, Norphanphoun C, Pem D, Phukhamsakda C, Sarma VV, Selcuk F, Senanayake IC, Shah S, Shu YX, Silva HVS, Su HL, Tavakol M, Thakshila SAD, Thiyagaraja V, Thongklang N, Tian Q, Tibpromma S, Tun ZL, Ulukapi M, Wang Y, Wannasawang N, Wijayawardene NN, Wimalasena SDMK, Xiao Y, Xiong YR, Yasanthika WAE, Li Q, Dai DQ (2023) Fungalpedia, an illustrated compendium of the fungi and fungus-like taxa. Mycosphere 14(1): 1835–1959. 10.5943/mycosphere/14/1/22

[B18] Kalichman J, Kirk PM, Matheny PB (2020) A compendium of generic names of agarics and *Agaricales*. Taxon 69(3): 425–447. 10.1002/tax.12240

[B19] Katoh K, Standley DM (2013) MAFFT multiple sequence alignment software version 7: improvements in performance and usability. Molecular Biology and Evolution 30(4): 772–780. 10.1093/molbev/mst010 PMC3603318 23329690

[B20] Khan J, Sher H, Khalid AN (2016) *Phaeocollybia pakistanica* sp. nov., the first representative of the genus from Pakistan. Mycotaxon 131(4): 889–896. 10.5248/131.889

[B21] Konrad P (1934) Le genre en mycologie. Schweizerische Zeitschrift für Pilzkunde 12: 175–178.

[B22] Kummer P (1871) Der Fuhrer in die Pilzkunde. Zerbst: C.Luppe.

[B23] Kuyper TW, Bas C (1986) (849) Proposal to conserve *Phaeocollybia* (*Cortinariaceae*, *Agaricales*). Taxon 740–743. 10.2307/1221637

[B24] Laber D (1991) Ergänzung zu Die europäischen Arten der Gattung *Phaeocollybia* und ihr Vorkommen im südlichen Schwarzwald. Zeitschrift für Mykologie 57: 109–116.

[B25] Liu PG (1995) Five new species of *Agaricales* from southern and southeastern Yunnan, China. Mycotaxon 56: 89–106. 10.5962/p.415914

[B26] Liu YJ, Whelen S, Hall BD (1999) Phylogenetic relationships among ascomycetes: Evidence from an RNA polymerase II subunit. Molecular Biology and Evolution 16(12): 1799–1808. 10.1093/oxfordjournals.molbev.a026092 10605121

[B27] Matheny PB, Curtis JM, Hofstetter V, Aime MC, Moncalvo JM, Ge ZW, Yang ZL, Slot JC, Ammirati JF, Baroni TJ, Bougher NL, Hughes KW, Lodge DJ, Kerrigan RW, Seidl MT, Aanen DK, Denitis M, Daniele GM, Desjardin DE, Kropp BR, Norvell LL, Parker A, Vellinga EC, Vilgalys R, Hibbett DS (2006) Major clades of *Agaricales*: a multilocus phylogenetic overview. Mycologia 98(6): 982–995. 10.1080/15572536.2006.11832627 17486974

[B28] Miller MA, Pfeiffer W, Schwartz T (2010) Creating the CIPRES Science Gateway for infer-ence of large phylogenetic trees. 10.1109/GCE.2010.5676129

[B29] Norvell LL (1998a) Observations on development, morphology and biology in *Phaeocollybia*. Mycological Research 102(5): 615–630. 10.1017/S0953756297005431

[B30] Norvell LL (1998b) The biology and taxonomy of Pacific Northwest species of *Phaeocollybia* Heim (*Agaricales*, *Cortinariaceae*). University of Washington.

[B31] Norvell LL (2000) *Phaeocollybia* in western North America. I. The *Phaeocollybia kauffmanii* complex. Canadian Journal of Botany 78(8): 1055–1076. 10.1139/b00-082

[B32] Norvell LL (2002) *Phaeocollybia* in western North America 3: New species *P. phaeogaleroides* and *P. rifflipes*, with notes on the *Phaeocollybia festiva* complex. Mycotaxon 81: 95–112.

[B33] Norvell LL, Exeter RL (2007) *Phaeocollybia* in western North America 5: *P. ochraceocana* sp. nov. and the *P. kauffmanii* complex. Mycotaxon 102: 315–332. 10.5962/p.414489

[B34] Norvell LL, Exeter RL (2008) *Phaeocollybia* of Pacific Northwest North America. 10.5962/bhl.title.121387

[B35] Norvell LL, Redhead SA (2000) *Phaeocollybia* in western North America 2: the vernal *P. pleurocystidiata* sp. nov. and *P. carmanahensis* reconsidered. Mycologia 92(5): 984–991. 10.1080/00275514.2000.12061242

[B36] Norvell LL, Exeter RL, Gordon M, Redhead SA (2010) Species concepts in a molecular age: the *Phaeocollybia* waltz. Abstract in IMC9: The biology of fungi. Oxford abstract disc for 1750 IMC9 delegates.

[B37] Norvell LL, Exeter RL, Gordon M, Mullineux ST, Redhead SA (2022) *Phaeocollybia chefensis* sp. nov. and new synonyms for *P. rifflipes*, *P. rufotubulina*, and *P. tibiikauffmanii*. Mycotaxon 137(1): 1–30. 10.5248/137.1

[B38] Nylander JA, Ronquist F, Huelsenbeck JP, Nieves-Aldrey J (2004) Bayesian phylogenetic analysis of combined data. Systematic Biology 53(1): 47–67. 10.1080/10635150490264699 14965900

[B39] Quélet L (1872) Les champignons du Jura et des Vosges. Impr. et lithographie de H. Barbier.

[B40] Rannala B, Yang Z (1996) Probability distribution of molecular evolutionary trees: a new method of phylogenetic inference. Journal of Molecular Evolution 43: 304–311. 10.1007/BF02338839 8703097

[B41] Redhead SA, Malloch DW (1986) The genus *Phaeocollybia (Agaricales)* in eastern Canada and its biological status. Canadian Journal of Botany 64(6): 1249–1254. 10.1139/b86-172

[B42] Redhead SA, Smith AH (1986) Two new genera of agarics based on *Psilocybe corneipes* and *Phaeocollybia perplexa*. Canadian Journal of Botany 64(3): 643–647. 10.1139/b86-082

[B43] Ronquist F, Teslenko M, van der Mark P, Ayres DL, Darling A, Höhna S, Larget B, Liu L, Suchard MA, Huelsenbeck JP (2012) MrBayes 3.2: efficient Bayesian phylogenetic inference and model choice across a large model space. Systematic Biology 61(3): 539–542. 10.1093/sysbio/sys029 PMC3329765 22357727

[B44] Singer R (1970) *Phaeocollybia* (*Cortinariaceae*–*Basidiomycetes*). Flora Neotropica 4: 1–13.

[B45] Singer R (1986) The *Agaricales* in Modern Taxonomy. 4^th^ edn. Koeltz Scientific Books, Koenigstein, 981 pp.

[B46] Smith AH (1937) New and unusual Agarics from the western United States. Mycologia 29(1): 45-59. 10.1080/00275514.1937.12017177

[B47] Smith AH (1957) A contribution toward a monograph of *Phaeocollybia*. Brittonia 9(4): 195–217. 10.2307/2804723

[B48] Smith AH, Trappe JM (1972) The higher fungi of oregon’s cascade head experimental forest and vicinity—I. The genus *Phaeocollybia (Agaricales)* and notes and descriptions of other species in the *Agaricales*. Mycologia 64(5): 1138–1153. 10.1080/00275514.1972.12019358

[B49] Stamatakis A (2014) RAxML version 8: a tool for phylogenetic analysis and post-analysis of large phylogenies. Bioinformatics 30(9): 1312–1313. 10.1093/bioinformatics/btu033 PMC3998144 24451623

[B50] Tang SM, Zhao G, Niu KY, Li RY, Yu FM, Karunarathna SC, Li L, Hyde KD, Su XJ, Luo ZL (2025) Species diversity of edible mushrooms I—Four new *Laccaria* species from Yunnan province, China. Journal of Fungi 11: 189. 10.3390/jof11030189 PMC11942694 40137227

[B51] Vaidya G, Lohman DJ, Meier R (2011) SequenceMatrix: concatenation software for the fast assembly of multi‐gene datasets with character set and codon information. Cladistics 27(2): 171–180. 10.1111/j.1096-0031.2010.00329.x 34875773

[B52] Vilgalys R, Hester M (1990) Rapid genetic identification and mapping of enzymatically amplified ribosomal DNA from several *Cryptococcus* species. Journal of Bacteriology 172(8): 4238–4246. 10.1128/jb.172.8.4238-4246.1990 PMC213247 2376561

[B53] Wang L, He SY, Lambert C, Zhu YG, Zhang JL, Yang Y, Li WL, Zhou HM, Zhao CL (2026) Comprehensive morphological and phylogenetic analyses of *Hymenochaetales (Basidiomycota)* unveil one new genus and twenty-six novel wood-inhabiting species from southwestern China. Persoonia 56: 328–380. 10.3114/persoonia.2026.56.05 PMC13409181 42524241

[B54] Wei TZ, Fu SZ, Qu PP, Yao YJ (2010) *Phaeocollybia purpurea* (*Cortinariaceae*), a new species from Wuyishan, China. Mycotaxon 113(1): 423–430. 10.5248/113.423

[B55] Wei TZ, Lv HM, Yao YJ (2012) Literature Review of *Phaeocollybia* species in China. Journal of Fungal Research 10(3): 173–176.

[B56] White TJ, Bruns T, Lee SJWT, Taylor J (1990) Amplification and direct sequencing of fungal ribosomal RNA genes for phylogenetics. PCR Protocols: A Guide to Methods and Applications 18(1): 315–322. 10.1016/B978-0-12-372180-8.50042-1

